# Why UK medical students change career preferences: an interview study

**DOI:** 10.1007/s40037-020-00636-7

**Published:** 2020-12-23

**Authors:** Amit Singh, Hugh Alberti

**Affiliations:** 1 Masters in Medical Education degree programme, Newcastle University, Newcastle upon Tyne, UK; 2 School of Medical Education, Newcastle University, Newcastle upon Tyne, UK

**Keywords:** Career, Preference, Change, Decision-making

## Abstract

**Introduction:**

Medical career preferences are influenced by a multitude of factors. Currently several specialties are undergoing recruitment problems; we must develop our understanding of medical career decision-making to ensure the production of an appropriate workforce. We aimed to explore the changing career preferences of students during medical school, to better understand this.

**Methods:**

This was an interpretivist, qualitative study. Data were collected through semi-structured interviews with seven final-year students to explore why their career preferences had changed during medical school. Transcripts of these interviews were thematically analysed.

**Results:**

Three overarching themes emerged from the analysis: The ‘influence of medical school’, ‘perceived suitability to specialty’ and ‘belonging and fitting in’. A thematic map captured the participants’ perceptions on why their preferences had changed, with major influences echoing existing research. However, novel findings included participants’ personalities and enthusiasm changing over time, the need for a ‘sense of belonging’ and participants defining the term ‘variety’ uniquely, perceiving their current specialty preference to match their definition.

**Discussion:**

This was an original, in-depth study on changing career preferences, which is an ill-defined subject within the literature. Analysis revealed preferences changed for a variety of medical school, personal and specialty reasons, leading to the construction of an updated model of medical career decision-making. Medical career preference remains a dynamic and ever-evolving phenomenon, influenced by an intricate interplay of internal and external factors. An understanding of this is crucial for future workforce planning.

**Electronic supplementary material:**

The online version of this article (10.1007/s40037-020-00636-7) contains supplementary material, which is available to authorized users.

## Introduction

Medical career preferences are influenced by multiple factors. Currently, several specialties are undergoing recruitment problems and there is an urgent need to develop an understanding of medical career decision-making to ensure an appropriate future workforce. However, the complex interplay of these factors can make predicting career destinations difficult, particularly when complicated by individuals’ preferences changing, and specialty choice trends changing over time.

Arguably, the purpose of medical education is to ensure there are sufficient numbers of competent, safe doctors in each specialty to meet the needs of a population and deliver effective care. As such, it is crucial for medical educators to understand how medical graduates make decisions about their career choice.

A plethora of factors have been found to influence career preferences, with career decisions being described as a ‘dynamic, complex and multifactorial issue’ [1]. The Bland-Meurer model of medical career choice suggests for medical specialty choice, ‘students rationally match perceptions of different specialties’ characteristics against a checklist of personal needs to form their specialty preference’ [2].

Medical schools themselves have been found to play a role in affecting career choice, with several studies showing a variation between medical schools in terms of graduate career intentions [3–6]. Additionally, personal characteristics have been found to influence career choice, with gender having a significant impact. [3, 4, 6–8]. Other factors include ethnicity [6], age of entry into medical school [9], personal values [10, 11] and arguably personality traits [12, 13].

Medical students have personal needs that they wish to fulfil in their professional lives [1]. These career needs can be split into three categories: ‘personal needs’, ‘societal needs’ and the ‘expectation of others’, with ‘personal needs’ tending to be the most influential [1]. Personal needs include expected salary, work/life balance, labour content, intellectual satisfaction, variety and lifestyle [4, 6, 7]. The term ‘societal needs’ is linked to ‘altruism’, whereby individuals feel a desire and moral responsibility to help and serve society, something that has correlated with specialties such as general practice [1, 14]. ‘Expectation of others’ refers to the opinions of others shaping career preferences, such as parental expectation [1, 15].

Speciality preference is known to change in around a quarter of students during medical school [16, 17] and final speciality destination has been matched to stated speciality preference after one year (65%) and three years (79%) [18] suggesting medical school is a vital period. Few studies have investigated change in preference during medical school, but factors that have been proposed as potentially causing a change include badmouthing of specialties, gender, ethnicity, medical school, positive clinical exposure, salary and lifestyle factors [4, 19–21].

Despite numerous literature reviews about career choice in medicine, critical analyses [22, 23] of the conceptual and methodological quality of the literature suggest a lack of appropriate theoretical research frameworks and a lack of longitudinal study designs. There are no qualitative studies to our knowledge that explore the phenomenon of changing career preferences in medical students. We aimed to explore the changing career preferences of students during medical school, in order to develop a better understanding of medical career decision-making.

## Methods

Due to the complex nature of the phenomena under consideration, an interpretivist approach was taken to thoroughly explore the perceptions of each student and understand their own individual context. Participants were found using purposive sampling. An internet advertisement for this study was placed on a student page of a UK medical school, specifying that final year students who had changed their preference over the course medical school were needed. At this stage it was not asked at what point their preference had changed.

Participation was voluntary and no incentives for taking part were offered. All final year students were informed of the study by email and were invited to volunteer if they had a clear speciality preference; they were asked to declare their previous and current speciality preference. Those students who had different previous and current speciality preferences were invited to participate and interviews continued until data saturation was reached.

Face-to-face, semi-structured interviews were conducted to explore students’ perceptions of factors that had shaped their change of preference of specialty during medical school using an interview schedule built on the previous literature on career choice factors. Informed, written consent was obtained. All interviews were transcribed verbatim by a professional transcription service. Transcripts were then analysed using Braun and Clarke’s inductive approach to thematic analysis [24] following the six steps of familiarising oneself with the data, generating initial codes, searching for themes, reviewing themes, defining and naming themes and producing the report. Initial analysis was undertaken by AS with discussion with HA after each step. Ethical approval was granted by the University Research Ethics committee.

Rigour was maintained using a research log, keeping a reflexivity journal to help us better consider our own thoughts and biases, member checking (transcripts were sent to participants to verify data) and regular review of emerging themes by both authors.

## Results

There were seven participants (two male and five female) with an array of changing career preferences as shown in Tab. 1. Inductive thematic analysis of the seven interviews derived an initial 94 codes, which was then refined to 58 codes by combining similar codes, such as ‘intellectual satisfaction’ and ‘intellectual stimulation’, or by removing themes that were deemed superficial or that were considered not related to the research question. This led to the formation of themes and subthemes (see Fig. 1 in Electronic Supplementary Material [ESM]).

**Table 1:** Participants

No	Specialty Preference on Medical School Entry	Current Specialty Preference	Gender
1	Psychiatry	Palliative care	F
2	Emergency medicine (A&E)	Surgery (plastics)	M
3	Pathology	Gastroenterology	F
4	Paediatrics	Emergency medicine or Surgery (orthopaedics)	F
5	Obstetrics & gynaecology (O&G)	General practice	F
6	Surgery (cardiothoracic)	Paediatrics or General practice	M
7	Surgery (cardiothoracic)	Cardiology	F

Consistent with hermeneutic phenomenology, the findings of the interviews have been presented as an interpretive narrative [25]. Quotes have been used as examples to demonstrate these themes and narratives and have been quoted exactly as spoken.

### Theme 1A: Influence of clinical teachers

Experiences at medical school was found to be key to the participants, mainly in terms of encountering a range of doctors and being exposed to different specialties through placements and the curriculum. These experiences served to dissuade them from their initially preferred specialty, attract them to their newly preferred specialty or act as some combination of the two. Participants reported both positive and negative experiences of doctors during their time at medical school with equal ability to impact on career preferences.

*The surgeons would happily be like ‘right can you please come and assist me I don’t have an assistant’ and the whole time they’re talking me through ‘this is what you do’. … so that’s probably the biggest thing, people just taking the time to show you things and talk to you which I feel like has a positive influence on me.* (Participant 2 (Accident & Emergency [A&E] → Surgery))

For many participants, the positive impact of a doctor was so profound that they were labelled as ‘role models’, whom the participants expressed a desire to emulate in their future professional lives.

*I just thought that the way he interacted with them, and knew everything about every single one of them, I just thought that he was the kind of doctor that I’d like to be*. (Participant 4 (Paediatrics → A&E/Surgery))

Negative experiences of doctors were described to be equally powerful in how much they could deter someone from their original specialty preference. This was found to occur in a variety of manners including direct methods such as discouragement and warnings, or indirectly through witnessing objectionable attitudes.

*I had doctors (in O&G) making some really unpleasant comments about women and it made me really cross, just making comments like if they were overweight … it was vile. That’s kind of what put me off actually*. (Participant 5 (Obstetrics & Gynaecology [O&G] → GP))

*I guess it’s like when you have a teacher that you don’t really like as much therefore don’t like the subject as much*. (Participant 1 (Psychiatry → Palliative Care))

All participants reported hearing negative comments and being aware of stereotypes about an array of specialties, but particularly general practice and surgery. The influence that these comments had on the participants was varied. For some, they had no effect at all or it was merely an annoyance, but for others, these comments were a direct reason for being deterred from a specialty, including the specialty they initially preferred.

*The influence that senior clinicians have upon particularly very junior medical students, I think that they themselves underestimate that, because I know for a fact that negative comments about certain specialties … put me off those specialties … in the first two years of medicine I was probably more receptive to those comments*. (Participant 2 (A&E → Surgery))

### Theme 1B: Influence of the curriculum and clinical placements

Participants viewed the curriculum and their placements as valuable sources of information regarding specialties and their nature. Lectures and the contents of the curriculum provided some participants with a clearer idea about the theory of a specialty and what it actually involves, for better or worse, but were not able to spark interest on their own.

*Obviously the first two years are kind of pre-clinical, but I sort of realised that a lot of pathology wasn’t forensic and when I saw the other side of it, I was like ‘this is definitely not what I want to do’.* (Participant 3 (Pathology → Gastroenterology))

One participant perceived themselves to be so unprepared by the curriculum for their initial choice of specialty that they had felt forced to change their preference.

*At [this medical school] we get taught anatomy in the first and second years but I don’t think its anywhere near as thorough as other universities … when it came to getting your clinical experience in third year and going into surgery, I felt very overwhelmed because I didn’t feel like I was possibly prepared enough for it, I didn’t feel I knew my anatomy well enough. It made you feel like ‘oh I probably couldn’t do this’.* (Participant 7 (Surgery → Cardiology))

Placements were used to confirm or refute preferences, which is particularly true for the ‘student selected components’ (SSCs), where the students were able to choose three specialties in which to spend six weeks in each during their fourth year of study. All participants referred to this experience when it came to evaluating specialties. Some who were still quite fluid in their preferences viewed experiencing their placements as a ‘process of elimination’.

*On the wards, the difference is it’s not like an academic interest it’s like ‘do I like this specialty, do I like the pace, the environment?’ and again you can either like tick or cross it off. I guess it just teaches you what you’re interested in versus what you’re not*. (Participant 1 (Psychiatry → Palliative Care))

*The opportunity that medical school gives you in regard to the SSCs and stuff, I think is a fantastic opportunity, because it allows you to go beyond the scope of the curriculum and look at things that would interest you personally.* (Participant 2 (A&E → Surgery))

A fundamental aspect of experiencing the curriculum and placements was the opportunity for participants to compare their expectation of a speciality to its reality. In many instances, a discrepancy between expectation and reality was cited as being a key reason for being dissuaded from a specialty. Reasons for these differences included enjoying the theoretical nature of a specialty but not its practical side, being unaware of the actualities of a specialty and having a misrepresented view of a specialty from pre-medical schoolwork experience.

*I definitely thought of surgery as the be all and end all like it was what I wanted to do and it’s what I’m going to work and do it, but I think it was such a dream that the reality of it was so different, and I think it shook me when doing it as a medical student and seeing it as a medical student, it was so differen*t. (Participant 6 (Surgery → Paediatrics/GP))

Conversely, participants also described the opposite, where they found that they had held inaccurate opinions of a specialty that were changed by experiencing them during medical school, resulting in the participant changing their career preference.

*I just found that I really liked the atmosphere in theatres, it wasn’t what I thought that obviously is depicted in films, or whatever, it’s all very serious, everyone was quite open to having a laugh and it’s a nice friendly atmosphere, you work in a team, everyone knows their job and they stick to it and I just really liked being around that.* (Participant 4 (Paediatrics → A&E/Surgery))

### Theme 2A: Enthusiasm and innate interest

Enjoyment of a specialty was a major influence on whether participants wanted to pursue it as a career. Consistently, participants struggled to pinpoint why they enjoyed a specialty, but described it was ‘innate’ and ‘natural’. Moreover, this enthusiasm for a specialty was not permanent for all participants and was subject to change over the course of medical school.

*It was just third year when every single time I woke up and went into my paediatric placement I had a massive smile on, like I was really excited, I really enjoyed it.* (Participant 6 (Surgery → Paediatrics/GP))

Participants discussed what they appreciated about specialties and what they considered ‘intellectual stimulation’. This was unique to the individual and was given different meanings by participants. Several described the enjoyment they took from doing technical, ‘hands-on’ work, contrasting with others who were more fulfilled by diagnosing and problem-solving. Some participants perceived this to change over time, consequently affecting their preference of specialty.

*I really now enjoy the process of actually working out what’s wrong with someone and actually being the problem-solver. With surgery you’re basically doing what someone else has told you to do. Whereas you’re actually working out, you’re taking the little pieces, you’re putting it all together. So that’s what I now value more as a doctor*. (Participant 7 (Surgery → Cardiology))

### Theme 2B: Personal characteristics

A multitude of personal characteristics was reported by participants to influence career preferences, including gender and personality. Gender was discussed by several female participants who perceived their gender to influence specialties they considered to be potential paths, particularly regarding surgical careers. Surgery was frequently described as being male-dominated and a ‘boys club’, which some participants did not feel welcome to.

*… with surgery, it makes me scared to go on a surgical placement because I feel really intimidated by them and I guess that is because they have a reputation, and I guess it put me off surgery all together.* (Participant 3 (Pathology → Gastroenterology))

Conversely, Participant 4, whose current preference is surgery, viewed this attitude as a motivator and ‘responsibility’ for their preference. This ‘moral responsibility’ was further alluded to by several participants as motivators behind their career preferences. These contexts included an overall lack of GPs and a lack of attention paid to mental health provision.

*Potentially it [surgery being male-dominated] made me want to do it more in terms of you couldn’t change those stereotypes and I think to have more women in a specialty that is very, very male dominated at the moment would only be a good thing*. (Participant 4 (Paediatric → A&E/Surgery))

*The fact that we need GPs so desperately, as a country crying out for GPs is also a major thing that make me want to do it. I feel like that might put some people off but for me that puts me on, that makes me want to do it more because I think it’s just the right thing to do*. (Participant 5 (O&G → GP))

A number of participants described their desire to see an impact as a result of their work as an influence on their career preferences, with one participant citing it as a reason for being put off their initial career preference.

*… whereas in a lot of aspects of mental health, I feel like there is very slow progress and whilst you might improve one thing for example, the person who’s got loads of problems, you improve one thing like that’s great but I’d always be like ‘well I haven’t really fixed everything have I?’ like I’d always be like ‘there’s more I could have done’.* (Participant 1 (Psychiatry → Palliative Care))

### Theme 2C: Perceptions of specialty characteristics

Perceptions of a specialty and its characteristics were described as an influence on career preferences. Participants also voiced a desire for ‘variety’ in their future career, despite this meaning something different for each participant. Some considered a varied working day as satisfying their need for variety, for example, switching between clinics and practical activities. For others ‘variety’ meant dealing with an array of diseases and body systems or having a flexible career path. The participants perceived their current specialty preference to match their definition of ‘variety’.

*I liked that it’s got quite a good mix of a lot of different things, so there’s from like alcoholic liver disease through to all the gut problems, there’s a little bit of everything, which I like. And I like that they have endoscopy, because I don’t want to be a surgeon, but that’s like a hands-on procedure, I like the idea of that as well.* (Participant 3 (Pathology → Gastroenterology))

Participants articulated their preferences about the pace of work. Some prefer fast-paced environments due to their ‘exciting’ nature, whereas others would rather work in a speciality where they have more time to focus on their patients and think through their decisions.

*I like working in an environment where you can work slowly but thoroughly so you tick all the boxes.* (Participant 1 (Psychiatry → Palliative Care))

Perceptions about patient interactions and relationships were mentioned as being key influences on enjoying a specialty and on changing preferences. Participants described how they were discouraged from some specialities, such as surgery and psychiatry, by a perceived lack of patient contact.

*Another thing that’s really put me off surgery is the lack of patient contact in terms of face-to-face communication, which is something that I’ve really learnt to value as a student because I think [this medical school] is very, very good at teaching communication skill, very good as focusing on your patient/doctor interaction and building that rapport. And I think you do lose that somewhat in surgery.* (Participant 7 (Surgery → Cardiology))

For several participants, the competitiveness of a specialty had little to no influence on their preference of that specialty, whether it was a very competitive or undersubscribed specialty. However, one participant cited competition as a major cause of their change of preference.

*Yeah, a huge influence [competitiveness of surgery]. I go home and read about it and you see, I don’t know, one year there’s only six jobs available for cardio-thoracic surgery training programme and you’re like ‘that’s impossible to get into, am I going to be one of the six?’ Why not go into something that is actually a lot easier to get into and it means I can actually enjoy the process of getting into it as opposed to work so hard to jump through the hoops to get to where I want, if that makes sense?* (Participant 6 (Surgery → Paediatrics/GP))

Several participants described a change in their professional outlooks during medical school, on subjects such as ‘work/life balance’, which subsequently impacted on their preference of specialty. These participants were those who had initial preferences of A&E or surgery.

*I guess it’s just maturing really through medical school because you realise ‘what kind of specialty do you want to do?’ Do you want something that will mean you’ve got not that great social hours that you might be called into work at any time?* (Participant 2 (A&E → Surgery))

While participants acknowledged salary and financial incentives, as well as length of training, as something they had considered, this was not found to be something that had a great deal of sway in their career preferences changing. Other incentives such as personal satisfaction and enjoyment were valued more than remuneration.

*The financial incentives do attract me definitely but if I had to rank them of how high up they are of my decision I’d probably say out of ten, it’s probably seven or eight, like one being top priority and ten being lowest priority.* (Participant 2 (A&E → Surgery))

### Theme 3: Belonging and fitting in

Participants perceived that they felt as if they ‘fit’ into their new specialty. Much of this sense of ‘belonging’ came from the essence of both Themes 1 and 2, such as positive experiences as well as an innate interest in the subject and a sentiment that they were personally ‘suited’ to the specialty. For several participants, this feeling was ineffable and was simply something inherent and innate.

*… Gastro was the one where I was like ‘yes, I love this’ and I just felt at home*. (Participant 3 (Pathology → Gastroenterology))

In terms of changing preferences, some participants felt they were unsuited to their initial specialty preference, whereas others felt that they simply suited their current preference of specialty better. A variety of reasons were given for not feeling suited to their initial preference, such as a mismatch of personalities or attitudes, a lack of enjoyment of the day-to-day work or not feeling at peace within the working environment of the specialty.

*Palliative care, everyone was super nice and I’m not saying that’s not the case in mental health but I just felt that they were my people … for me it’s a priority to make sure that the patients are happy and it’s nice to share that opinion and passion with other people working at the same place.* (Participant 1 (Psychiatry → Palliative Care))

Participants could envisage themselves working within the specialty in the future. This was the result of a complex interplay of core elements from Themes 1 and 2: experiences, characteristics of a specialty and personality. This was referred to by all participants and was considered to be influential, with one participant describing this as the most significant reason for changing their specialty preference.

*… the experiences that I’ve had with doctors, Obs and gynae doctors and GPs has been the single biggest factor [in changing preference] I would say because I can see myself being a GP, but I can’t see myself being an Obs and gynae doctor now having seen what I’ve seen. It’s not what I want anymore*. (Participant 5 (O&G → GP))

## Discussion

Our original, in-depth study has demonstrated that students’ career preferences were found to change for a variety of medical school, personal and specialty reasons. The findings have suggested a modification to the Bland-Meurer model of speciality choice to incorporate the theme of ‘belonging and fitting in’.

### Influence of experiences and placements

Experiences at medical school were the strongest influence in changing career preferences. This occurred through a combination of negative experiences of initially preferred specialties and positive experiences of their current preferences, derived from the curriculum, clinical teachers and clinical placements.

Placements were seen by participants as opportunities to confirm or refute preferences through learning more about specialties, with some undergoing a ‘process of elimination’. This has been described within the literature reported [1, 6, 15] with medical career selection being equated to a ‘process of hypothesis generation and testing’ [26]. Placements provided opportunity for engaging with doctors, who were found to be both the best and worst advertisement for their own specialty [1, 21, 27–29]. This study adds to this by providing insight into some of the perceived factors that make a good role model and what students look for in their clinical teachers to inspire them. Negative comments made by clinicians about specialties other than their own were heard by all participants but were found to have varying degrees of influence. For some, this influence was strong enough to deter them from specialties, including specialties that they held initial enthusiasm for, but for others the impact was marginal. Negative comments and undermining have been found to be widespread in medical schools and the workplace [19, 20, 30]. It is not known whether the reverse of this holds true: whether or not students are deterred from specialties that talk negatively about other specialties. It would be plausible that this may be the case for some, but this was not described within this study.

### Perceived suitability to speciality

Participants perceived themselves to be suited or unsuited to a specialty for a variety of reasons: the student’s enthusiasm and innate interest in a specialty, personal characteristics and their perceptions of specialty characteristics. Although enthusiasm for a subject has previously been found to be a strong influence [6], a new finding of our study was that this enthusiasm was not necessarily permanent, and faded in some cases as the participants progressed through medical school, despite being ‘innate’ in nature.

Regarding personal characteristics, gender is an influence, with several female participants highlighting how they were dissuaded from surgical careers due to its male-dominated nature as described previously [8, 29, 31, 32]. Interestingly, one female participant considered becoming a female surgeon as a ‘responsibility’ in order to change gender stereotypes, using this as a motivation to pursue the career. This is not reflected within the literature and perhaps warrants further investigation. Responsibility was also alluded to by other participants in their reasons for preferring their new specialty, referring to ‘societal’ and ‘altruistic’ responsibilities, as described previously [1, 11, 12, 14].

Personality played a key role in several changing preferences in this study, with some participants feeling that their personality meant they were better suited to certain specialties than others. These tended to revolve around what participants enjoy and look for in a specialty, some taking satisfaction from taking a practical approach to work, others who enjoyed problem-solving and diagnosing and others who appreciated the building of patient relationships [12, 13, 33]. In addition, several participants in this study described how their personality had evolved over the course of medical school, leading them to alter their preferences based on these personality changes, even from being a ‘hands-on’ person to a ‘person-oriented’ person. This finding may provide an indication that these aspects of personality are subject to change and can subsequently affect career preferences. Additionally, it is possible that a student could initially self-assess their own personality incorrectly. The ‘personality change’ mentioned above could be the product of the better personal understanding that comes with the self-assessment and self-reflection that occurs during medical school, which occurs during the formative years of young adulthood for many students.

Perceptions of specialty characteristics were reported to influence specialty preferences. A key finding of this study was that all participants expressed a desire for variety in their future career, and all felt that they had found this within their current specialty preference. Studies have found variety to be an influence on career preference [1], but little has been described about what this actually means to an individual.

Some participants changed their specialty preference as result of perceived differences between their outlook and the lifestyle of their initially preferred specialty. This was particularly true for those who had an early preference of A&E or surgery. This ‘maturation’ has been described within the literature, where students are described to, at first, be purely influenced by their perception of the content of work but are later likely to become more pragmatic through experience and consider other lifestyle factors [1, 21, 34].

Competition, salary and financial incentives, and length of training were all factors participants had considered, but overall were not a strong influence on their changes in preferences, though previous findings on this vary [6]. It is important to remember that this study only looked at medical students from the United Kingdom. The weighting of competition and salary may be vastly different in other countries, which have their own individual working landscapes for doctors.

### Belonging and fitting in

All participants perceived that they fit in with their current preference of specialty and felt ‘at home’. One participant stated ‘I felt that they were my people’ when describing staff within their preferred specialty. Equally, several participants described how this was not true for their early specialty choice, leading to a change in preference.

Participants felt that they ‘belonged’ if they perceived their personality to ‘match’ the specialty and if they appreciated the working lifestyle of the specialty, both of which stemmed from positive experiences. Other participants were unable to express why they felt this way, just that it was ‘inherent’. This sense of belonging along with perceiving that they are suited to the specialty and experiences throughout medical school all contribute to the participants being able to ‘envisage’ themselves in their preferred specialty, the ‘final destination’ in preferring a specialty while at medical school. This incorporates elements of the previous two themes: the student can only reach this point by progressing through medical school, experiencing different specialties and then processing the wealth of information to evaluate which specialty they wish to pursue.

### Updated career choice model

We have sought to address the shortcomings of previous literature on career change by addressing the theoretical underpinnings and mapping our findings to a known theoretical research framework.

The findings of this study agree with and map accurately to the components described in the Bland-Meurer Model, with the addition of a new concept: ‘belonging and fitting in’. We would recommend that it should mapped in addition to the existing components, as shown in Fig. 2 (see ESM, Fig. 2).

Other career theories such as the social cognitive career theory [34] would support our confirmatory findings of self-efficacy (Subtheme 2B) and outcome expectations (2C), and career self-determination theory [35] would support our novel additional finding of relatedness (Theme 3, ‘belonging and fitting in’).

### Strengths and limitations

Our study has produced a data set in an area that has not been well-studied, using a congruous methodology and filling a gap in the literature. This has led to the formation of an updated model. The interviewer and lead author is a relative research novice but input and guidance was provided by the second author, an experienced qualitative researcher and supervisor. A limitation of this study is the small sample size used—7 students out of a possible 300 final year medical students—and it is unknown how many of the cohort changed their career preference during their time at medical school. However, the purpose of this qualitative research was to explore the realities of medical students and their career preferences to help to provide a deeper and better understanding of these decisions rather than to seek a single overarching ‘truth’ as to why these decisions are made. Furthermore, a key participant was missing: the student whose preference shifted from general practice to another specialty. This proved to be an impossible find and it is unknown whether such a student existed within this cohort. Potential recall bias and only using students from one medical school in one country are further acknowledged limitations.

### Implications

Our findings are potentially transferable to other contexts and we would recommend that:

- Clinicians are made aware of the role modelling influence they can have on medical career choice. This is already well-described within the literature. However, the findings of this study further stress this.
- A zero-tolerance policy of specialty undermining and ‘negative comments’ be implemented as a method of maintaining professional standards.
- Measures are considered to address the potential gender disparity in surgery in the UK.

In terms of future research, we would recommend:

- Further research to explore and examine the sense of ‘belonging and fitting in’ that these participants have described.
- Further research on how and why personalities and enthusiasm change over time in the context of medical school, and how this subsequently affects career preferences.
- Further qualitative research into what makes a ‘positive’ or ‘negative’ clinical experience, which could provide more information about modifiable factors that can be changed by medical schools to improve the quality of the placements provided.

## Conclusion

This was an original study that produced in-depth findings on changing career preferences, a subject described poorly within the literature. Career preferences of medical students were found to change for a variety of reasons within three broad themes of the ‘influence of medical school’, ‘perceived suitability to specialty’ and ‘belonging and fitting in’. The first two themes are already described in an existing model of medical career decision-making. However, the exclusion of this study’s third theme from the existing model led to the formation of an updated version to incorporate this. Career preference remains a dynamic, complex and ever-evolving phenomenon, influenced by an intricate interplay of internal and external factors. Medical career decision-making is an important field of work if we are to ensure that medical schools are producing an appropriate workforce to meet the present and future demands of our healthcare system.

## Caption Electronic Supplementary Material

Interview Schedule

Fig. 1: Themes and subthemes

Fig. 2: Updated Bland-Meurer model of medical career choice
