# Supplementary material for: Why UK medical students change career preferences: an interview study
Source: Perspect Med Educ. 2020 Dec 23;10(1):41–9. doi: 10.1007/s40037-020-00636-7 (PMC7809071; doi:10.1007/s40037-020-00636-7)
Supplement: Supplementary file 1 — Interview Schedule [file 40037_2020_636_MOESM1_ESM.docx]

Interview Schedule

1. Setting the scene
   - Introduce and explain the study
   - Reassure participants that information is confidential and there are no right or wrong answers, that it is about the participants’ viewpoint
   - Honest answers are the most valuable
   - Explain audio recording again, obtain written consent
2. What was your preference of specialty coming into medical school?
   - Why/Expand?
   - Media? Work experience? Family? Personal experience of illness?
3. How has that preference changed since you have come to medical school?
4. When did the preference change? Gradually or suddenly?
5. Why has that preference changed?
   - Were any of the throng influences stronger than others?
   - Expand
6. What put you off your initial choice of specialty?
7. What has attracted you to your current choice of specialty?
8. How do you think your personality affects your preferences?
   - Do you feel more or less suited to some specialties?
9. How has your time at medical school shaped your career preference?
   - Experience? What about it?
   - Curriculum?
   - Role models?
   - Negative comments/stereotypes?
10. What characteristics do you value in your current preference of specialty?
    - Were they responsible for your changing preference?
    - Interest in the subject?
    - Length of training
    - Competition
    - Financial incentives
    - Working conditions
    - Patient contact
    - Specialist vs. generalist
    - Reputation/hierarchy
    - Work/life balance
    - Research
11. What has been the biggest influence on your changing career preference?
12. What put you off other specialties?
13. Is there anything that we haven’t discussed so far about what has influenced your career intentions?
14. Conclude – thank participant, ask if they have any questions.
