# Supplementary figures and images for: Why UK medical students change career preferences: an interview study
Source: Perspect Med Educ. 2020 Dec 23;10(1):41–9. doi: 10.1007/s40037-020-00636-7 (PMC7809071; doi:10.1007/s40037-020-00636-7)

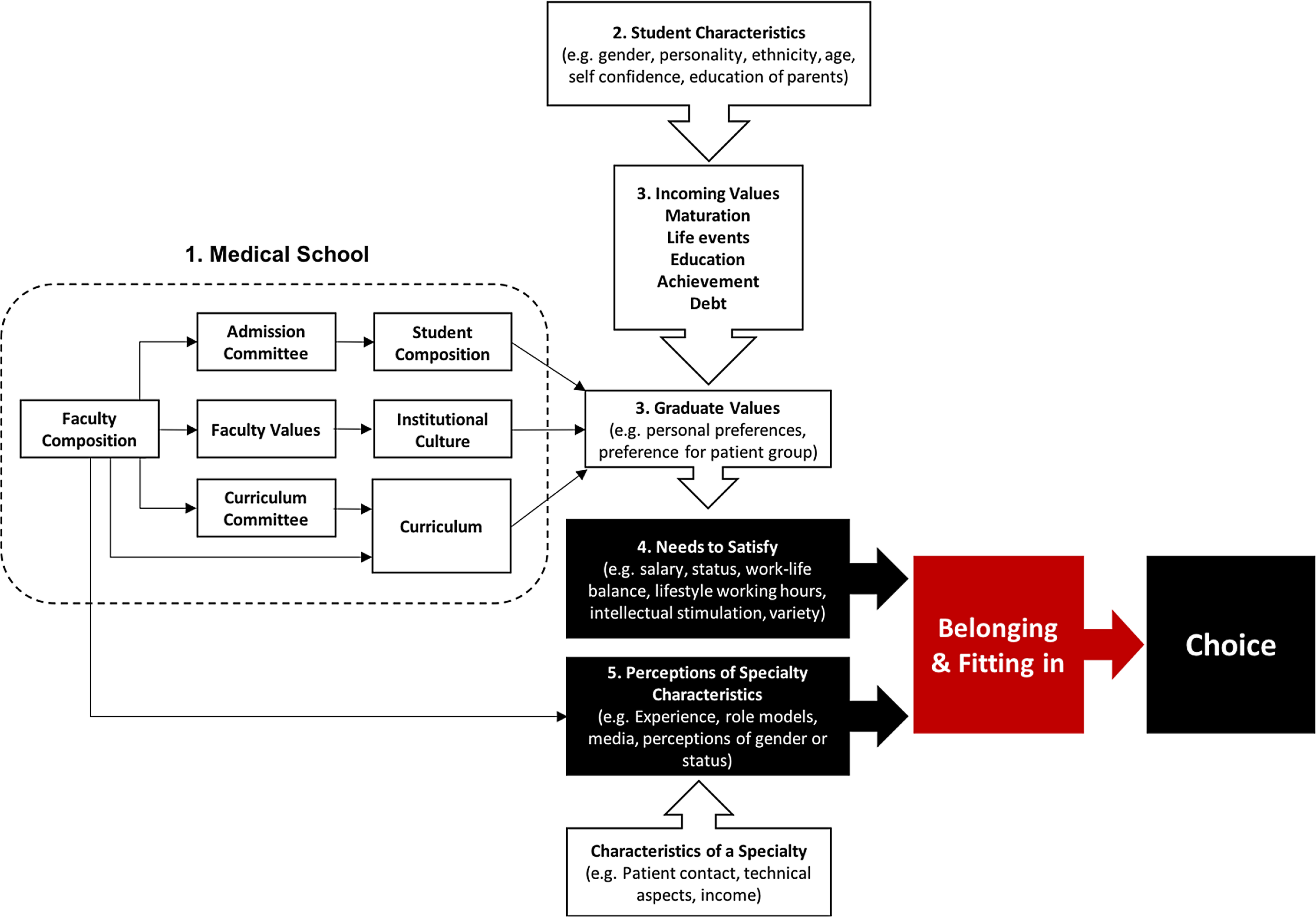

Supplement: Supplementary file 3 — Fig. 2: Updated Bland-Meurer model of medical career choice [file 40037_2020_636_MOESM3_ESM.tif]
